# Macrolide-Resistant *Bordetella pertussis* in Hong Kong: Evidence for Post-COVID-19 Emergence of *ptxP3*-Lineage MT28 Clone from a Hospital-Based Surveillance Study

**DOI:** 10.3390/microorganisms13081947

**Published:** 2025-08-20

**Authors:** Tsz-Yung Hui, Hayes Kam-Hei Luk, Garnet Kwan-Yue Choi, Sandy Ka-Yee Chau, Lok-Man Tsang, Cindy Wing-Sze Tse, Ka-Kin Fung, Jimmy Yiu-Wing Lam, Ho-Leung Ng, Tommy Hing-Cheung Tang, Edmond Siu-Keung Ma, Herman Tse, Sally Cheuk-Ying Wong, Vivien Wai-Man Chuang, David Christopher Lung

**Affiliations:** 1 Department of Pathology, Queen Elizabeth Hospital, Hong Kong SAR, China; daisy.ty.hui@ha.org.hk (T.-Y.H.); hayes.luk@ha.org.hk (H.K.-H.L.); wcy288@ha.org.hk (S.C.-Y.W.); 2 Department of Pathology, Hong Kong Children’s Hospital, Hong Kong SAR, China; cky603@ha.org.hk; 3 Department of Pathology, United Christian Hospital, Hong Kong SAR, China; chauky@ha.org.hk; 4 Department of Pathology, Kwong Wah Hospital, Hong Kong SAR, China; tlm477@ha.org.hk (L.-M.T.); tsewsc@ha.org.hk (C.W.-S.T.); 5 Department of Pathology, Pamela Youde Nethersole Eastern Hospital, Hong Kong SAR, China; fkk143@ha.org.hk (K.-K.F.); lyw543a@ha.org.hk (J.Y.-W.L.); 6 Department of Clinical Pathology, Tuen Mun Hospital, Hong Kong SAR, China; hl.ng1@ha.org.hk; 7 Department of Medicine, Queen Elizabeth Hospital, Hong Kong SAR, China; thc061@ha.org.hk; 8 Quality & Safety Division, Hospital Authority Head Office, Hong Kong SAR, China; chuangwm@ha.org.hk; 9 Infection Control Branch, Centre for Health Protection, Department of Health, Hong Kong SAR, China; edmond_sk_ma@dh.org.hk; 10 Department of Laboratory Medicine, Khoo Teck Puat Hospital, Singapore 768828, Singapore

**Keywords:** macrolide-resistant, *Bordetella pertussis*, pertussis, whooping cough

## Abstract

A post-COVID surge of *Bordetella pertussis* was observed globally. China has reported a high level of macrolide-resistant *Bordetella pertussis* (MRBP) in recent years; however, the epidemiology of MRBP in Hong Kong remains unknown. We retrieved archived *B. pertussis* isolates from respiratory samples collected at five regional public hospitals in Hong Kong between 2015 and 2024 and tested their minimum inhibitory concentration (MIC) for macrolides and other non-macrolide antibiotics using the Etest method. All isolates were also subjected to whole genome sequencing for genotypic resistance, Multi-locus Antigen Sequence Typing (MLST) and Multi-locus Variable Number of Tandem Repeat Analysis (MLVA) typing. Twenty-nine isolates of *B. pertussis* were included in the study. All isolates demonstrating phenotypic macrolide resistance harbored the A2047G mutation while showing low MIC to trimethoprim-sulfamethoxazole, doxycycline, levofloxacin, piperacillin-tazobactam and meropenem. In 2023 and 2024, 100% were MRBP and all belonged to the MT28 clone with the *ptxP3* antigenic type. The MRBP isolates in Hong Kong were phylogenetically related to those from mainland China during the same period. There was no obvious correlation between macrolide resistance and clinical presentation, laboratory findings, management and outcome. Phylogenetic analysis suggests that MRBP isolates in Hong Kong and mainland China are closely related.

## 1. Introduction

Pertussis, also known as whooping cough, is a highly contagious bacterial infection that was a leading cause of childhood illness and death in the pre-vaccine era. It classically presents in young infants with the characteristic paroxysmal cough, which may be lead to feeding difficulties, respiratory failure and even death in severe cases [1]. In the 1940s, pertussis caused three times more infant deaths than measles, mumps, chicken pox, rubella, scarlet fever, diphtheria, poliomyelitis and bacterial meningitis combined [2]. Global vaccination campaigns started in the 1950s with whole-cell vaccines, and later switched to acellular vaccines in a routine schedule in 1990s [3]. Despite these efforts, it was estimated that pertussis accounted for around 160,000 deaths worldwide in the year 2014 alone [4].

The pertussis-containing vaccine was first introduced in Hong Kong in 1957 [5]. Under the current Hong Kong Childhood Immunization Program, children are given three primary doses of pertussis-containing vaccine at two, four and six months and three booster doses from 18 months onwards. Acellular pertussis toxoid-containing vaccines haven been used since 2006 [6]. The immunization coverage rate has been over 95% for many years. In response to rising infant pertussis cases in Hong Kong, one dose of pertussis-containing vaccine in the second or third trimester is now offered to pregnant women as part of routine antenatal care since 2020, in the form of dTap (diphtheria (reduced dose), tetanus and acellular pertussis (reduced dose)) [5]. In mainland China, pertussis-toxoid acellular vaccine is administered at three, four, five and 18 months [7]. Vaccine coverage is reported at 99% [8]. The maternal pertussis vaccination is currently not integrated into healthcare practices in mainland China. A study shows that only 36% of expectant mothers intended to receive the pertussis-containing vaccine in a province in mainland China [9].

Despite the implementation of the childhood vaccination program and maternal vaccination, a recent upsurge of pertussis has been reported globally after the relaxation of non-pharmacological interventions for coronavirus disease-2019 (COVID-19) [10,11,12]. Data from the World Health Organization (WHO) show that the global incidence of pertussis increased from 3.6 million in 2021 to 22.8 million in 2023, nearing the pre-pandemic level [13]. A similar increase has been observed in Hong Kong [1]. The rise in incidence of whooping cough is most notable in vaccinated individuals and older children [14,15]. It has been proposed that vaccine escape of *B. pertussis* is due to the antigenic shift from the *ptxP1* to *ptxP3* subtype and the acquisition of macrolide resistance [16].

Macrolide resistance in *B. pertussis* is most commonly caused by an A-to-G mutation at position 2047 of the Sanger Center sequence of the *B. pertussis* 23s rRNA gene [17]. The mutation affects the macrolide binding site in the 23S rRNA component of the 50S ribosomal subunit, preventing macrolide inhibition on peptide elongation. There are three copies of the 23S rRNA gene in *B. pertussis*, but it was expected that mutation in at least two copies would be sufficient to confer resistance [17]. Macrolide-resistant *Bordetella pertussis* (MRBP) was first reported in the United States in 1994 [18]. The prevalence of MRBP in North America, Europe, Southeast Asia and Australia remained low at 0–13% from 1967 to 2020 [18], and has only sporadically been reported since, including one isolate recently reported from France [19]. In contrast, since the first report of MRBP in Shandong in mainland China in 2011, its prevalence in the major cities of mainland China, including Beijing and Shanghai, has been observed to reach up to 90–100% after the COVID-19 pandemic [15,20,21]. Pertussis toxin promoter (*ptxP*) is one of the antigenic subtypes included in the Multi-locus Sequence Typing (MLST) scheme historically used to study shifts in *B. pertussis* populations. The variant ptxP3 is traditionally believed to enhance the production of toxins [22]. Previous studies showed that MRBP in China was mostly linked to the *ptxP1* allele [21,23]. In 2007, mainland China introduced the acellular diphtheria–tetanus–pertussis (DTaP) vaccine which comprises strains with the *ptxP1* antigen [24]. The *ptxP3*-MRBP antigen has emerged sporadically in mainland China since 2017 and was found to be prevalent in Shanghai in 2021. This means that the *ptxP3* strain likely holds a competitive advantage over the ptxP1 strain under vaccine-driven selection pressure [25].

Hong Kong was experiencing a similar upsurge of pertussis in 2024 [26]. Although in close geographical proximity to mainland China, the prevalence and circulating antigenic genotype of MRBP in Hong Kong remained unknown. Azithromycin is currently the antibiotic recommended by local guidelines for pertussis treatment and post-exposure prophylaxis (PEP) [27], similar to the practice in the United States, United Kingdom and Australia where MRBP prevalence has remained low [28,29,30,31]. However, macrolides are ineffective against MRBP, permitting further transmission [28,30]. Trimethoprim-sulfamethoxazole is the alternative antibiotic choice, especially against MRBP. This retrospective study aims to investigate the epidemiology and genetic evolution of MRBP in Hong Kong.

## 2. Materials and Methods

### 2.1. Strain Recovery and Identification

All archived clinical *B. pertussis* isolates from respiratory samples collected during patient care at five regional public hospitals (Queen Elizabeth Hospital, United Christian Hospital, Tuen Mun Hospital, Kwong Wah Hospital and Pamela Youde Nethersole Eastern Hospital) in Hong Kong during 2015–2024 were retrospectively retrieved. Archived bacterial isolates stored at −70 °C in Microbank 2D (Pro-lab diagnostics; Richmond Hill, ON, Canada) were subcultured onto Regan–Lowe agar (Oxoid CM0119; Thermo Fisher, Basingstoke, UK) in an ambient moist chamber at 35 °C for up to 7 days. Bacterial speciation was reconfirmed by matrix-assisted laser desorption/ionization time-of-flight mass spectrometry (MALDI-TOF MS, IVD library version: DB-11758 MSP; Bruker, Bremen, Germany). Only one isolate will be retrieved for analysis for an individual pertussis case of close contact.

### 2.2. Antimicrobial Susceptibility Testing

The minimum inhibitory concentration (MIC) of three macrolides (erythromycin, azithromycin, clarithromycin), trimethoprim-sulfamethoxazole, doxycycline, levofloxacin, piperacillin-tazobactam and meropenem were determined after 72 h of incubation at 35 °C using MIC test strips (Liofilchem, Copenhagen, Denmark) and interpreted as previously described [32]. There is no standardized method for susceptibility testing of *B. pertussis*; however, the MICs of erythromycin against susceptible isolates were previously reported to range from 0.02 to 0.12 μg/mL [33].

### 2.3. Detection of A2047G Mutation

*B. pertussis* colonies were inoculated into 1.5 mL nuclease free water to create a suspension of 1.0 McFarland standard and heated to 100 °C. A total of 200 μL of the suspension was then added to 200 μL of MagNA Pure Bacterial Lysis Buffer (Roche, Basel, Switzerland) and MagNA Pure extraction was carried out according to the manufacturer’s instructions [34]. The extracted DNA was subjected to A2047G mutation detection by polymerase chain reaction-based sequencing of the 23S rRNA gene, as previously described [35].

### 2.4. Whole Genome Sequencing

Genomic DNA from *B. pertussis* isolates was extracted using DNeasy Blood & Tissue Kit (Qiagen, Hilden, Germany), followed by library preparation using Illumina DNA Prep (Illumina, San Diego, CA, USA). Sequencing of the pooled library was performed using a MiSeq sequencer (Illumina) with paired-end 300 bp reads, as previously described [36]. Briefly, the low-quality bases in the paired-end reads were filtered and trimmed using Trimmomatic v0.39 [37]. Quality-trimmed reads were then de-novo assembled using SPAdes v3.15.3 and Ragout v2.3 for the reference-assisted scaffolding [38,39]. Single nucleotide polymorphism (SNP) analysis was performed using Snippy v4.60 (https://github.com/tseemann/snippy (accessed on 13 August 2024)). *B. pertussis* strain Tohama I (Genbank accession no. NC_002929.2) was selected as the reference genome. A maximum likelihood phylogeny was constructed from the SNP data using IQ-TREE v2.1.1 with a K2P with ascertainment bias correction. The whole-genome sequencing project has been deposited in GenBank (accession numbers SAMN48174304-32).

### 2.5. Multi-Locus Sequence Typing (MLST) and Virulence Gene Analysis

The whole-genome sequencing (WGS) data was used to determine the multi-locus sequence type of each isolate. Allele numbers and sequence types (STs) were assigned by querying the Bordetella database in the BIGSdb-Pasteur platform (https://bigsdb.pasteur.fr/bordetella/ (accessed on 13 August 2024)). The presence and sequence variants of key virulence genes (ptxP, ptxA, ptxC, prn, fim2, fim3, tcfA, fhaB and bscI) were also determined from the WGS data using the Bordetella database in the BIGSdb-Pasteur platform.

### 2.6. Multi-Locus Variable Number of Tandem Repeat Analysis (MLVA) Typing

MLVA typing was performed according to a protocol previously described [40].

### 2.7. Retrieval of Clinical Data

Demographic data of the patients, including age, gender, isolation source and date, symptoms and signs of presentation, investigation results, clinical progress and outcome were retrieved by reviewing electronic medical records in the Hospital Authority Clinical Management System.

### 2.8. Consumption of Macrolides

Data on consumption of macrolides were obtained from the Department of Health in Hong Kong. The department collected the annual wholesale supply data from all registered drug wholesalers in Hong Kong. This served as a good proxy of antimicrobial consumption for the whole territory of 7.3 million people. Consumption of macrolides and other antimicrobials from 2015 to 2024 were analyzed and presented as Defined Daily Dose (DDD) and DID (DDD per 1000 Inhabitants per Day) as the units adopted by the World Health Organization (WHO). Version 2024 of the DDD values was adopted for the calculations.

### 2.9. Statistical Analysis

Statistical analysis was performed using SPSS version 24 (SPSS, Chicago, IL, USA). Statistical significance for the clinical severity of diseases due to MRBP and macrolide-sensitive *B. pertussis* (MSBP), *ptxP1* and *ptxP3* subtype, was determined by the Mann–Whitney U test and Fisher’s Exact Test. The consumption of macrolides over the surveillance period was tested for any statistical change using linear regression.

## 3. Results

**Figure 1:**
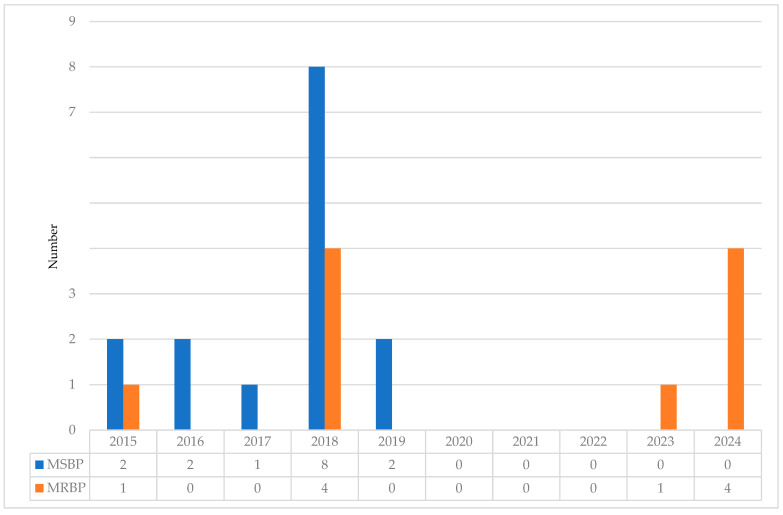
Macrolide resistance of *Bordetella pertussis* in Hong Kong, 2015–2024. MSBP, macrolide-sensitive *Bordetella pertussis*; MRBP, macrolide-resistant *Bordetella pertussis*.

Twenty-nine clinical *B. pertussis* isolates were recovered between January 2015 and May 2024 and included in our study. Macrolide-resistance was identified in 34.5% (10/29). The first MRBP was detected in 2015, and its prevalence reached 33% (4/12) in 2018. In 2023 and 2024, after the end of COVID-19 pandemic was declared by the World Health Organization [41], MRBP became dominant and now accounts for all of the tested isolates (1/1 in 2023, 4/4 in 2024) (Figure 1).

Twenty-five (25/29, 86.2%) of the cultured *B. pertussis* isolates were isolated from symptomatic cases, and the remaining four (13.8%) were isolated from close contacts who retrospectively reported mild upper respiratory tract infection symptoms (Table 1). The median age of our patients was three months, and 72.4% (21/29) were infants under six months old who would not have completed their primary immunization course against pertussis according to the local schedule. A majority (93.1%, 27/29) of patients had cough, but only 10.3% (3/29) exhibited the classical symptom of paroxysmal cough, while 13.8% (4/29) demonstrated wheezing. *B. pertussis* infection resulted in cyanosis in 31.0% (9/29), pneumonia in 17.2% (5/29) and respiratory failure in 13.8% (4/29) of patients, while 6.9% (2/29) of patients required mechanical ventilation and 10.3% (3/29) required supportive nasogastric feeding. All cases presenting with cyanosis, pneumonia, respiratory failure and requiring mechanical ventilation were infants under 6 months of age. The median white blood cell, lymphocyte and C reactive protein of the patients were 15.15 × 10^9^/L, 10.40 × 10^9^/L and 0.6 mg/L, respectively. One (3.5%) of the patients had recent use of a third-generation cephalosporin, while none had recently used a macrolide group antibiotic. All patients were treated with a macrolide group antibiotic, but one patient (case 25) was later switched to trimethoprim-sulfamethoxazole due to treatment failure and subsequent detection of phenotypic resistance. All patients survived and recovered after a median 4 days of hospital stay (Table 1).

**Figure 2:**
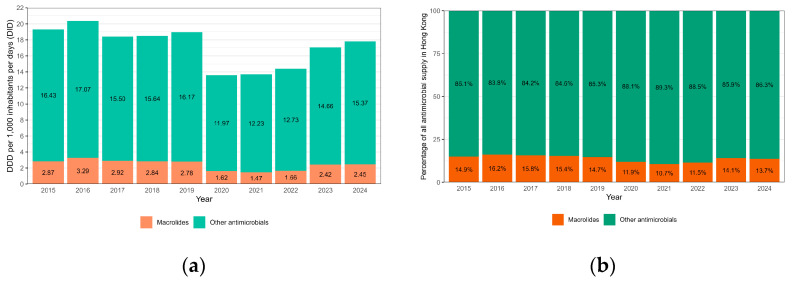
(**a**) Wholesale supply of antimicrobials in Hong Kong, 2015–2024, measured in daily defined dose per 1000 inhabitants; (**b**) Wholesale supply of antimicrobials in Hong Kong, 2015–2024, measured in percentage. Linear regression: *p* = 0.06.

Clinically, there was no obvious correlation between macrolide resistance and clinical presentation, laboratory findings, disease severity, management and outcome in our cohort (Table 1). Travel history to mainland China within 30 days was noted in 40% (4/10) of the patients infected with MRBP (Table 1). The other clinical data have been summarized in Table 1. Figure 2a shows the supply of macrolides and other antimicrobials in Hong Kong over the study period. The proportion of macrolides among all antimicrobials fluctuated between 14.7% and 16.2% from 2015 to 2019 before it dropped slightly during the COVID-19 period from 2020 to 2022, ranging from 10.7% to 11.9% (Figure 2b). Overall, there was no statistically significant change in the consumption pattern of macrolides from 2015 to 2024.

Macrolide resistance in *B. pertussis* is associated with the A2047G mutation in the 23S rRNA gene [35]. Although standardized breakpoints for detecting macrolide resistance in *B. pertussis* have not been established by the European Committee on Antimicrobial Susceptibility Testing (EUCAST) or the Clinical and Laboratory Standards Institute (CLSI), all *B. pertussis* isolates with the A2047G mutation in our study exhibited MICs of ≥256 μg/mL when tested with all three macrolides (azithromycin, erythromycin, clarithromycin) using the MIC test strip method. All isolates, irrespective of macrolide susceptibility, showed low MIC to all other tested antibiotics (Table 2). The MICs of individual isolates are summarized in Appendix A Table A1.

**Figure 3:**
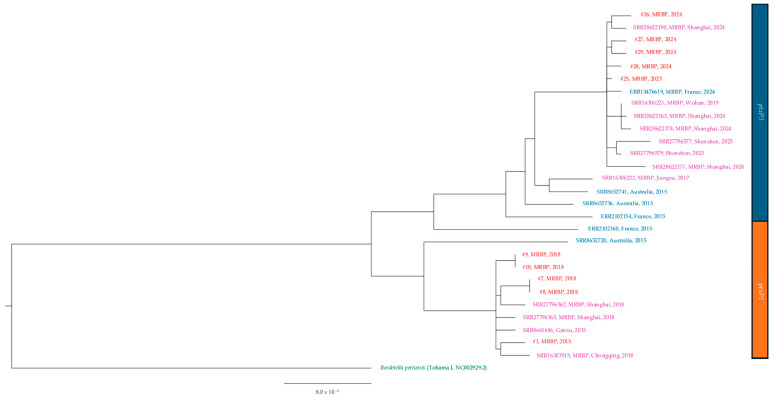
Phylogenetic tree based on the complete genome of macrolide-resistant *Bordetella pertussis* isolates and other isolates of the same period. In red, isolates from study; in purple, isolates from mainland China; in blue, isolates from other countries; in green, reference strain. #, isolate; MRBP, macrolide-resistant *Bordetella pertussis; ptxP1*, pertussis toxin promoter 1; *ptxP3*, pertussis toxin promoter 3.

**Figure 4:**
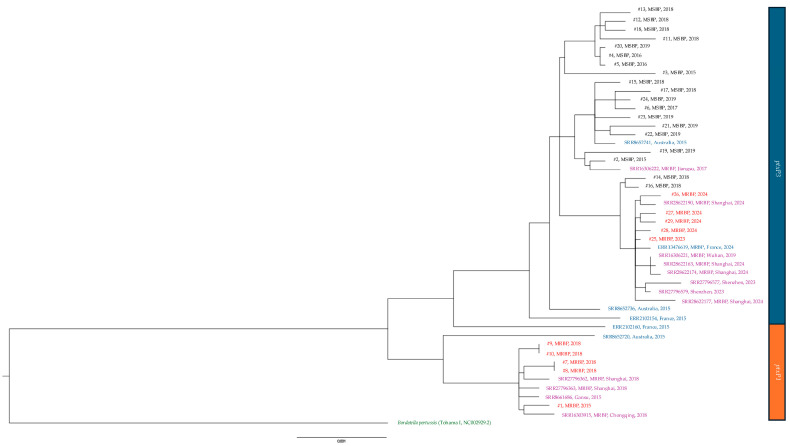
Phylogenetic tree based on the complete genome of macrolide-sensitive and macrolide-resistant *Bordetella pertussis* isolates and other isolates of the same period. In red, MRBP from study; in black, MSBP from study; in purple, MRBP from mainland China; in blue, MRBP from other countries; in green, reference strain. #, isolate; MSBP, Macrolide-sensitive *Bordetella pertussis*; MRBP, macrolide-resistant *Bordetella pertussis*; *ptxP1*, pertussis toxin promoter 1; *ptxP3*, pertussis toxin promoter 3.

All the 29 isolates were subjected to WGS analysis. Based on the results obtained, before 2019, all MSBP harbored *ptxP3/ptxA1/ptxC2/prn2/fim2-1/fim3-1/tcfA2/fhaB1/bscI2* and all MRBP harbored *ptxP1/ptxA1/ptxC1/prn1/fim2-1/fim3-1/tcfA2/fhaB3/bscI1*. After 2019, all MRBP harbored *ptxP3/ptxA1/ptxC2/prn150/fim2-1/fim3-1/tcfA2/fhaB1/bscI2*. The molecular typing results of the isolates are listed in Table 3, and all isolates after 2023 were MT28 (Table 3). No pertactin-deficient isolates were found in the current study. The phylogenetic relationship between our isolates, isolates from mainland China and worldwide are shown in Figure 3 and Figure 4.

## 4. Discussion

This is the first report to describe the molecular characteristics and macrolide resistance of *B. pertussis* in Hong Kong. MRBP was detected as early as 2015 in Hong Kong and was demonstrated to have reached a prevalence of 100% after the COVID-19 pandemic in our cohort. The COVID-19 pandemic was also observed to be a critical timepoint for the transformation and epidemiology of MRBP in the major cities of mainland China, such as in Shanghai (36.4% in 2016 to 97.2% in 2022) [16], Tianjin (94.44% in 2012–2017) [42], Shenzhen (46.8% in 2015–2017) [43], Hunan (49% in 2017) [44] and Xi’an (79.3% in 2018–2020) [45].

Previous genotypic studies show that the rising macrolide resistance and shifting virulence genes of *B. pertussis* were largely influenced by the overuse of macrolides in mainland China [46]. In Hong Kong, the apparent rise in MRBP was observed despite a fairly stable consumption of macrolides during the study period. The slight drop in macrolide usage during the COVID-19 pandemic was probably due to reduced respiratory infections, for which macrolides were often prescribed especially in outpatient settings [47]. Instead, we observed that a higher proportion (40%, 4/6) of the MRBP cases in our study had a recent travel history to mainland China. Our latest MRBP isolates in 2023 and 2024 exhibited the same genotype and virulence-associated allelic profiles as the recent *ptxP3*/*prn150* MR-MT28 strain circulating in mainland China [8,15]. Our genome analysis shows that the macrolide-resistant isolates in our cohort are closely associated with MRBP from mainland China during the same time period (Figure 3). The MRBP collected in 2024 in France (ERR13476619) was genotypically and phylogenetically related to isolates from China [19]. The above suggests that the MRBP seen in Hong Kong could have been introduced from mainland China.

Consistent with previous studies, we demonstrated no significant difference in clinical outcomes between MSBP and MRBP infections despite the use of ineffective antimicrobials in the latter group of patients [15]. Despite similar clinical outcomes, MRBP-infected patients can act as a persistent source of transmission due to use of ineffective antibiotics [15]. Macrolides were shown to be ineffective for eradication of MRBP in the nasopharynx in previous studies, with clearance of 51.2% and 74.2% on day 7 and 14 after treatment, respectively, with the longest carriage time of up to 81 days after initial detection [48]. The selection of MRBP and its prolonged shedding with the use of macrolides can facilitate its transmission in the community.

The clinical significance of the allelic divergence of *B. pertussis* remains controversial. Strains containing *ptxP3* were believed to be more virulent as they produced more pertussis toxins than *ptxP1* strains in some previous studies, but infections by *ptxP3* strains were previously noted to be milder and were more likely to affect older, vaccinated individuals [14,22,49]. When stratifying the clinical outcomes according to pertussis promotor toxins, we demonstrated that our *ptxP3*-containing *B. pertussis* strains resulted in significantly higher hospitalization rates compared to *ptxP1*-containing strains, but other clinical outcomes were not significantly different (Appendix A Table A2). Our study also demonstrated a shift in the dominant virulence gene *prn*. All MSBP harbored the *prn2* allele and all MRBP harbored the *prn1* allele before 2019 (Table 3). After 2019, all *B. pertussis* isolates were macrolide-resistant and harbored *prn150*. The shift to *prn150* was also observed in other studies as a result of divergence from the vaccine strain [24]. The clinical significance of *prn150* has not been described.

### 4.1. Future Directions

Without effective antimicrobials to eradicate the nasopharyngeal carriage of MRBP, prolonged asymptomatic shedding can act as a continuous source leading to community outbreaks. This is an urgent call to review local treatment and post-exposure prophylaxis strategies by considering alternatives to macrolides to control pertussis. Both trimethoprim-sulfamethoxazole and doxycycline are shown to be effective in vitro for killing *B. pertussis* in previous studies [50]. Several fluoroquinolones, including levofloxacin, which was also tested in our study, showed good in vitro activity against *B. pertussis* and achieved concentrations in respiratory secretions that were well above their MICs [51]. However, trimethoprim-sulfamethoxazole is not licensed for infants younger than 6 weeks old [30]. Tetracyclines were believed to cause dental staining and were previously avoided in children younger than 8 years old. Recent guidelines suggest that newer generation tetracyclines, e.g., doxycycline, is not likely to cause visible teeth staining or enamel hypoplasia in children younger than 8 years when administered for short durations [52]. Although current guidelines do not routinely recommend the use of levofloxacin in children younger than 18 years, it can be used in specific conditions when there are no alternatives, or when the drug is known to be effective for the specific situation [53]. For children requiring inpatient treatment, beta-lactam group antibiotics such as piperacillin-tazobactam or meropenem may be considered. The effectiveness of beta-lactam group antibiotics was demonstrated in previous in vivo studies, where 91.5% and 95.7% clearance of MRBP in the nasopharynx was achieved on day 7 and day 14 after treatment, respectively [48]. Cefoperazone-sulbactam was also demonstrated to result in satisfactory clinical clearance of *B. pertussis* in the nasopharynx [54]. These drugs can be safely used in infants but require intravenous access and hospitalization.

Despite the high prevalence of MRBP, the latest Chinese National Guidelines for Treatment and Post-exposure Prophylaxis for *B. pertussis* in 2024 still recommend azithromycin as the first-line treatment for whooping cough, but recommend hospitals with adequate resources to perform resistance testing for *B. pertussis* [55]. Second-line agents include trimethoprim-sulfamethoxazole or levofloxacin for infants older than 2 months and adults, and piperacillin-tazobactam or cefoperazone-sulbactam for those younger than 2 months [55]. The local guidelines in Hong Kong give similar recommendations [27]. Our latest data suggest that there is an imminent need for Hong Kong to take the lead to update its guidelines to control MRBP.

### 4.2. Call to Action

In the context of globalization, a comprehensive surveillance system for MRBP is necessary [56]. This includes a high index of suspicion for whooping cough even in older and vaccinated individuals, and a low threshold for testing. Cultures should be routinely performed for polymerase chain reaction (PCR) positive cases, and the isolate should be subjected to antimicrobial resistance testing and epidemiological study using molecular methods. Hong Kong, an international financial hub with up to 34 million international visitors in 2023 [57], can potentially disseminate MRBP to other geographical regions if the transmission of MRBP is not adequately controlled. Prompt actions to update guidelines with the use of non-macrolide antibiotics may be required to help contain MRBP and prevent regional and global spread.

### 4.3. Limitations

Only 6.7% (29/431) of pertussis case reported to the Centre for Health Protection in Hong Kong from 2015 to June 2024 could be included in the current study. Several factors contributed to the low inclusion rate. Firstly, the molecular method is the main avenue for diagnosis of pertussis in Hong Kong, while *B. pertussis* culture is only performed on PCR-positive patients from respiratory specimens taken after the PCR result is available [58]. As macrolides are often started empirically for highly suspicious cases before PCR results are available in practice, a per-nasal culture swab taken after the PCR positive result and empirical treatment may have a lower yield. Due to the retrospective nature of our study, some archived isolates were no longer available at the time of our study.

Additionally, a majority of the isolates (24/29) from our study were from children, with 72.4% being under 6 months of age. Unfortunately, data on maternal immunization against pertussis was not available from the electronic medical record. However, as the maternal immunization program was implemented only after July 2020, it is likely that none of the mothers of the infant cases would have received the pertussis vaccine before 2020.

Finally, *B. pertussis* infections in adults are often mild and less symptomatic, so diagnosing pertussis is challenging in adults and could be underrepresented in our cohort [59]. Only 1/5 of our isolates from adult patients presented symptomatically, while the rest were isolated by contact tracing.

## 5. Conclusions

This is the first study to describe the epidemiology and prevalence of MRBP in Hong Kong. The first MRBP isolate was detected in 2015, and after the COVID-19 pandemic its prevalence has reached 100%. MRBP isolates in Hong Kong showed phylogenetic relatedness to isolates in mainland China around the same period. All MRBP showed low MIC to non-macrolide antimicrobials, which should be considered as first-line agents for effective treatment and eradication of MRBP to prevent regional and global spread.

## Figures and Tables

**Table 1:** Clinical Symptoms, Laboratory Findings, Clinical Outcome and Management of 29 Cases with Culture-proven Pertussis—by A2047G mutation.

Characteristics	All Cases (*n* = 29)	With A2047G Mutation (*n* = 10)	Without A2047G Mutation (*n* = 19)	*p*-Value ^a^
Case/contact	25/4	7/3	18/1	NA
Age (years, median, IQR)	0.25, 0.08–0.5	0.33, 0.25–14.00	0.17, 0.05–0.42	NA
Travel history to mainland China within 30 days	6 (20.7%)	4 (40%)	2 (10.5%)	NA
Clinical symptoms—*n* (%)				
Paroxysmal cough	3 (10.3%)	2 (20%)	1 (5.3%)	NS
Fever	2 (6.90%)	2 (20%)	0 (0.0%)	NS
Cyanosis	9 (31.0%)	3 (30%)	6 (31.6%)	NS
Wheezing	4 (13.8%)	1 (10%)	3 (15.8%)	NS
Pneumonia	5 (17.2%)	3 (30%)	2 (10.5%)	NS
Respiratory failure	4 (13.8%)	1 (10%)	3 (15.8%)	NS
Laboratory findings—median (IQR)				
White blood cell (×10^9^/L) ^b^	15.15 (10.70–23.70)	14.12 (10.70–38.50)	15.25 (11.45–23.65)	NS
Lymphocyte (×10^9^/L) ^b^	10.40 (6.40–18.10)	10.10 (8.66–21.56)	10.55 (6.40–13.70)	NS
C reactive protein (mg/L) ^b^	0.60 (0.25–1.00)	1.08 (0.25–3.00)	0.30 (0.20–1.00)	NS
Clinical Outcome—*n* (%)				
Hospitalization	22 (75.9%)	7 (70%)	15 (78.9%)	NS
Length of hospital stay, median (inter-quartile range, days)	4.00 (3.00–6.00)	3.50 (0.00–6.00)	4.00 (3.00–6.00)	NS
Survival	29 (100%)	10 (100%)	19 (100%)	NA
Required oxygen therapy	4 (13.8%)	1 (10%)	3 (15.8%)	NS
Required mechanical ventilation	2 (6.90%)	0 (0.0%)	2 (10.5%)	NS
Required nasogastric feeding	3 (10.3%)	1 (10%)	2 (10.5%)	NS
Antibiotic therapy ^c^—*n* (%)				
Macrolide	28 (96.6%)	9 (90%)	19 (100%)	NS
Trimethoprim-sulfamethoxazole ^c^	1 (3.45%)	1 (10%)	0 (0.0%)	NS
Prior antibiotic usage ^d^—*n* (%)				
Third-generation cephalosporin	1 (3.45%)	0 (0.0%)	1 (7.1%)	NS
Macrolide	0 (0.0%)	0 (0.0%)	0 (0.0%)	NA

IQR, interquartile range; NA, not applicable; NS, not significant. ^a^ *p*-value was calculated with the Mann–Whitney U test for parameters including white blood cells, lymphocytes, C reactive protein and length of stay. The *p* value was calculated with Fisher’s Exact Test for parameters including paroxysmal cough, fever, cyanosis, wheezing, pneumonia, respiratory failure, hospitalization, survival, oxygen therapy, mechanical ventilation and nasogastric feeding. ^b^ Result of this parameter is not available for all cases. ^c^ One patient did not receive treatment, while one patient received macrolide followed by trimethoprim-sulfamethoxazole due to treatment failure. ^d^ Within two weeks.

**Table 2:** Phenotypic and Genotypic Test Results of 29 *Bordetella pertussis* Isolates Isolated from Hong Kong.

A2047G Mutation	Number	MIC_90_, MIC Range (μg/mL)							
		ERY	AZT	CLR	SXT	DOX	LEV	TZP	MEM
Detected	10	≥256, ≥256	≥256, ≥256	≥256, ≥256	1.05, 0.032–1.5	3, 1–3	0.263, 0.25–0.38	<0.016, <0.016	0.0971, 0.032–0.125
Not detected	19	0.19, 0.047–0.19	0.1002, 0.032–0.125	0.9, 0.38–2	1, 0.047–1	3, 1–4	0.38, 0.19–0.38	<0.016, <0.016	0.125, 0.008–0.125

MIC, minimum inhibitory concentration; ERY, erythromycin; AZT, azithromycin; CLR, clarithromycin; SXT, trimethoprim-sulfamethoxazole; DOX, doxycycline; LEV, levofloxacin; TZP, piperacillin-tazobactam; MEM, meropenem.

**Table 3:** Demographics, Genotypic, MLST and MLVA Results of 29 *Bordetella pertussis* Isolates Isolated from Hong Kong.

Isolate	Case/Contact	Sex	Age	Year	Travel History to Mainland China *	A2047G Mutation	Virulence Genes									MT	Biosample Accession Number
							*ptxP*	*ptxA*	*ptxC*	*prn*	*fim2*	*fim3*	*tcfA*	*fhaB*	*bscI*		
1	Case	F	4 m	2015	Y	D	*ptxP1*	*ptxA1*	*ptxC1*	*prn1*	*fim2-1*	*fim3-1*	*tcfA2*	*fhaB3*	*bscI1*	104	SAMN48174304
2	Case	F	4 m	2015	Y	ND	*ptxP3*	*ptxA1*	*ptxC2*	*prn2*	*fim2-1*	*fim3-1*	*tcfA2*	*fhaB1*	*bscI2*	26	SAMN48174305
3	Case	M	2 m	2015	N	ND	*ptxP3*	*ptxA1*	*ptxC2*	*Not found*	*fim2-1*	*fim3-1*	*tcfA2*	*fhaB1*	*bscI2*	27	SAMN48174306
4	Case	M	1 m	2016	N	ND	*ptxP3*	*ptxA1*	*ptxC2*	*prn2*	*fim2-1*	*fim3-1*	*tcfA2*	*fhaB1*	*bscI2*	27	SAMN48174307
5	Case	M	1 m	2016	N	ND	*ptxP3*	*ptxA1*	*ptxC2*	*prn2*	*fim2-1*	*fim3-1*	*tcfA2*	*fhaB1*	*bscI2*	27	SAMN48174308
6	Case	M	1 m	2017	N	ND	*ptxP3*	*ptxA1*	*ptxC2*	*prn2*	*fim2-1*	*fim3-1*	*tcfA2*	*fhaB1*	*bscI2*	27	SAMN48174309
7	Case	F	2 m	2018	N	D	*ptxP1*	*ptxA1*	*ptxC1*	*prn1*	*fim2-1*	*fim3-1*	*tcfA2*	*fhaB3*	*bscI1*	104	SAMN48174311
8	Contact	M	32 y	2018	N	D	*ptxP1*	*ptxA1*	*ptxC1*	*prn1*	*fim2-1*	*fim3-1*	*tcfA2*	*fhaB3*	*bscI1*	104	SAMN48174312
9	Contact	M	53 y	2018	N	D	*ptxP1*	*ptxA1*	*ptxC1*	*prn1*	*fim2-1*	*fim3-1*	*tcfA2*	*fhaB3*	*bscI1*	104	SAMN48174325
10	Contact	M	14 y	2018	N	D	*ptxP1*	*ptxA1*	*ptxC1*	*prn1*	*fim2-1*	*fim3-1*	*tcfA2*	*fhaB3*	*bscI1*	104	SAMN48174326
11	Case	F	1 m	2018	N	ND	*ptxP3*	*ptxA1*	*ptxC2*	*prn2*	*fim2-1*	*fim3-1*	*tcfA2*	*fhaB1*	*bscI2*	27	SAMN48174310
12	Case	F	3 m	2018	N	ND	*ptxP3*	*ptxA1*	*ptxC2*	*prn2*	*fim2-1*	*fim3-1*	*tcfA2*	*fhaB1*	*bscI2*	27	SAMN48174315
13	Case	M	1 m	2018	N	ND	*ptxP3*	*ptxA1*	*ptxC2*	*prn2*	*fim2-1*	*fim3-1*	*tcfA2*	*fhaB1*	*bscI2*	27	SAMN48174313
14	Case	F	1 m	2018	N	ND	*ptxP3*	*ptxA1*	*ptxC2*	*prn2*	*fim2-1*	*fim3-1*	*tcfA2*	*fhaB1*	*bscI2*	161	SAMN48174316
15	Case	M	1 m	2018	N	ND	*ptxP3*	*ptxA1*	*ptxC2*	*prn2*	*fim2-1*	*fim3-1*	*tcfA2*	*fhaB1*	*bscI2*	27	SAMN48174314
16	Case	M	1 m	2018	Y	ND	*ptxP3*	*ptxA1*	*ptxC2*	*prn2*	*fim2-1*	*fim3-1*	*tcfA2*	*fhaB1*	*bscI2*	28	SAMN48174317
17	Case	F	3 m	2018	N	ND	*ptxP3*	*ptxA1*	*ptxC2*	*prn2*	*fim2-1*	*fim3-1*	*tcfA2*	*fhaB1*	*bscI2*	27	SAMN48174324
18	Case	M	35 y	2018	N	ND	*ptxP3*	*ptxA1*	*ptxC2*	*prn2*	*fim2-1*	*fim3-1*	*tcfA2*	*fhaB1*	*bscI2*	27	SAMN48174318
19	Case	F	2 m	2019	N	ND	*ptxP3*	*ptxA1*	*ptxC2*	*prn2*	*fim2-1*	*fim3-1*	*tcfA2*	*fhaB1*	*bscI2*	27	SAMN48174319
20	Case	F	5 m	2019	N	ND	*ptxP3*	*ptxA1*	*ptxC2*	*prn2*	*fim2-1*	*fim3-1*	*tcfA2*	*fhaB1*	*bscI2*	27	SAMN48174329
21	Contact	F	58 y	2019	N	ND	*ptxP3*	*ptxA1*	*ptxC2*	*prn2*	*fim2-1*	*fim3-1*	*tcfA2*	*fhaB1*	*bscI2*	27	SAMN48174330
22	Case	F	2 m	2019	N	ND	*ptxP3*	*ptxA1*	*ptxC2*	*prn2*	*fim2-1*	*fim3-1*	*tcfA2*	*fhaB1*	*bscI2*	114	SAMN48174331
23	Case	F	6 m	2019	N	ND	*ptxP3*	*ptxA1*	*ptxC2*	*prn2*	*fim2-1*	*fim3-1*	*tcfA2*	*fhaB1*	*bscI2*	27	SAMN48174327
24	Case	M	42 y	2019	N	ND	*ptxP3*	*ptxA1*	*ptxC2*	*prn2*	*fim2-1*	*fim3-1*	*tcfA2*	*fhaB1*	*bscI2*	27	SAMN48174332
25	Case	M	3 m	2023	Y	D	*ptxP3*	*ptxA1*	*ptxC2*	*prn150*	*fim2-1*	*fim3-1*	*tcfA2*	*fhaB1*	*bscI2*	28	SAMN48174328
26	Case	M	3 m	2024	Y	D	*ptxP3*	*ptxA1*	*ptxC2*	*prn150*	*fim2-1*	*fim3-1*	*tcfA2*	*fhaB1*	*bscI2*	28	SAMN48174320
27	Case	M	2 m	2024	N	D	*ptxP3*	*ptxA1*	*ptxC2*	*prn150*	*fim2-1*	*fim3-1*	*tcfA2*	*fhaB1*	*bscI2*	28	SAMN48174321
28	Case	M	9 y	2024	Y	D	*ptxP3*	*ptxA1*	*ptxC2*	*prn150*	*fim2-1*	*fim3-1*	*tcfA2*	*fhaB1*	*bscI2*	28	SAMN48174322
29	Case	M	4 m	2024	N	D	*ptxP3*	*ptxA1*	*ptxC2*	*prn150*	*fim2-1*	*fim3-1*	*tcfA2*	*fhaB1*	*bscI2*	28	SAMN48174323

MLST, Multilocus Sequence Typing; MLVA, Multi-locus Variable Number of Tandem Repeat Analysis; M, male; F, female; m, month; y, year; Y, yes; N, no; D, detected; ND, not detected; *ptxP*, *Bordetella pertussis* promoter; *ptxA*, pertussis toxin A; *ptxC*, pertussis toxin C; *prn*, pertactin; *fim2*, fimbriae 2; *fim3*, fimbriae 3; *tcfA*; tracheal colonization factor A; *fhaB*, filamentous hemaggluttin; *bscI*, a type III secretion system gene. * within 4 weeks.

## Data Availability

The original contributions presented in this study are included in the article. Further inquiries can be directed to the corresponding author.

## Abbreviations

The following abbreviations are used in this manuscript:

MRBP	Macrolide-resistant *Bordetella pertussis*
MIC	Minimum inhibitory concentration
MLST	Multi-locus antigen sequence typing
MLVA	Multi-locus variable number of tandem repeat analysis
dTap	Diphtheria (reduced dose), tetanus & acellular pertussis (reduced dose)
COVID-19	Coronavirus disease-2019
WHO	World Health Organization
PEP	Post-exposure prophylaxis
DDD	Defined daily dose
MSBP	Macrolide-sensitive *B. pertussis*
EUCAST	European Committee on Antimicrobial Susceptibility Testing
CLSI	Clinical and Laboratory Standards Institute
PCR	Polymerase chain reaction

## Appendix A

**Table A1:** Patient Demographics, Phenotypic and Genotypic Testing Results of 29 Bordetella pertussis Isolates.

Isolate	Case/ Contact	Sex	Age	Year	Travel History to Mainland China *	A2047G Mutation	Minimum Inhibitory Concentration (μg/mL)							
							ERY	AZT	CLR	SXT	DOX	LEV	TZP	MEM
1	Case	F	4 m	2015	Y	D	≥256	≥256	≥256	0.38	2	0.25	<0.016	0.094
2	Case	F	4 m	2015	Y	ND	0.064	0.094	0.75	0.5	2	0.25	<0.016	0.125
3	Case	M	2 m	2015	N	ND	0.094	0.094	0.5	1	3	0.25	<0.016	0.094
4	Case	M	1 m	2016	N	ND	0.19	0.094	0.38	0.38	3	0.38	<0.016	0.094
5	Case	M	1 m	2016	N	ND	0.047	0.094	0.38	0.38	2	0.25	<0.016	0.064
6	Case	M	1 m	2017	N	ND	0.047	0.125	0.75	0.38	2	0.25	<0.016	0.094
7	Case	F	2 m	2018	N	D	≥256	≥256	≥256	0.5	2	0.25	<0.016	0.125
8	Contact	M	32 y	2018	N	D	≥256	≥256	≥256	0.032	1	0.25	<0.016	0.032
9	Contact	M	53 y	2018	N	D	≥256	≥256	≥256	0.064	2	0.25	<0.016	0.094
10	Contact	M	14 y	2018	N	D	≥256	≥256	≥256	0.38	2	0.25	<0.016	0.094
11	Case	F	1 m	2018	N	ND	0.094	0.094	0.75	0.75	2	0.25	<0.016	0.064
12	Case	F	3 m	2018	N	ND	0.19	0.094	0.75	0.5	2	0.25	<0.016	0.064
13	Case	M	1 m	2018	N	ND	0.125	0.094	0.5	0.094	2	0.25	<0.016	0.125
14	Case	F	1 m	2018	N	ND	0.064	0.064	0.75	0.5	2	0.38	<0.016	0.047
15	Case	M	1 m	2018	N	ND	0.19	0.064	0.75	0.25	2	0.25	<0.016	0.008
16	Case	M	1 m	2018	Y	ND	0.125	0.094	0.75	1.5	2	0.25	<0.016	0.064
17	Case	F	3 m	2018	N	ND	0.094	0.047	0.75	0.047	2	0.38	<0.016	0.047
18	Case	M	35 y	2018	N	ND	0.125	0.094	2	1	1.5	0.25	<0.016	0.094
19	Case	F	2 m	2019	N	ND	0.125	0.125	1.5	0.75	3	0.19	<0.016	0.125
20	Case	F	5 m	2019	N	ND	0.064	0.032	0.5	0.5	2	0.25	<0.016	0.094
21	Contact	F	58 y	2019	N	ND	0.094	0.032	0.5	0.38	3	0.38	<0.016	0.094
22	Case	F	2 m	2019	N	ND	0.094	0.032	0.5	0.25	2	0.38	<0.016	0.094
23	Case	F	6 m	2019	N	ND	0.064	0.047	0.75	0.047	1	0.19	<0.016	0.064
24	Case	M	42 y	2019	N	ND	0.094	0.064	0.75	1	4	0.25	<0.016	0.094
25	Case	M	3 m	2023	Y	D	≥256	≥256	≥256	0.5	3	0.25	<0.016	0.064
26	Case	M	3 m	2024	Y	D	≥256	≥256	≥256	1	3	0.25	<0.016	0.094
27	Case	M	2 m	2024	N	D	≥256	≥256	≥256	0.5	3	0.25	<0.016	0.094
28	Case	M	9 y	2024	Y	D	≥256	≥256	≥256	0.5	2	0.25	<0.016	0.094
29	Case	M	4 m	2024	N	D	≥256	≥256	≥256	1.5	2	0.38	<0.016	0.094

M, male; F, female; m, month; y, year; Y, yes; N, no; D, detected; ND, not detected; ERY, erythromycin; AZT, azithromycin; CLR, clarithromycin; SXT, trimethoprim-sulfamethoxazole; DOX, doxycycline; LEV, levofloxacin; TZP, piperacillin-tazobactam; MEM, meropenem. * within 4 weeks.

**Table A2:** Clinical Symptoms, Laboratory Findings, Clinical Outcome and Management of 29 Cases with Culture-proven Pertussis—by *ptxP*.

Characteristics	All Cases (*n* = 29)	*ptxP1* (*n* = 5)	*ptxP3* (*n* = 24)	*p*-Value	Odds Ratio ^a^	
					Estimate	95% CI
Clinical symptoms—*n* (%)						
Paroxysmal cough	3 (10.3%)	1 (20%)	2 (8.3%)	NS	NA	NA
Fever	2 (6.90%)	1 (20%)	1 (4.2%)	NS	NA	NA
Cyanosis	9 (31.0%)	2 (40%)	7 (29.2%)	NS	NA	NA
Wheezing	4 (13.8%)	1 (20%)	3 (12.5%)	NS	NA	NA
Pneumonia	5 (17.2%)	0 (0%)	5 (20.8%)	NS	NA	NA
Respiratory failure	4 (13.8%)	0 (0%)	4 (16.7%)	NS	NA	NA
Laboratory findings—median (IQR)						
White blood cell (×10^9^/L) ^b^	15.15 (10.70–23.70)	27.6 (16.40–38.80)	13.75 (10.70–23.65)	NS	NA	NA
Lymphocyte (×10^9^/L) ^b^	10.40 (6.40–18.10)	19.6 (10.10–29.10)	10.40 (6.40–13.70)	NS	NA	NA
C reactive protein (mg/L) ^b^	0.60 (0.25–1.00	0.91 (0.25–1.56)	0.60 (0.20–1.00)	NS	NA	NA
Clinical outcome—*n* (%)						
Survival	29 (100%)	5 (100%)	24 (100%)	NA	NA	NA
Hospitalization	22 (75.9%)	2 (40%)	21 (87.5%)	<0.05	0.10	(0.01, 0.83)
Length of hospital stay, median (interquartile range, days)	4.00 (3.00–6.00)	0.00 (0.00–6.00)	4.00 (3.00–5.50)	NS	NA	NA
Required oxygen therapy	4 (13.8%)	0 (0.0%)	4 (16.7%)	NS	NA	NA
Required mechanical ventilation	2 (6.90%)	0 (0.0%)	2 (8.3%)	NS	NA	NA
Required nasogastric feeding	3 (10.3%)	1 (20%)	2 (8.3%)	NS	NA	NA
Antibiotic therapy ^c^—*n* (%)						
Macrolide	28 (96.6%)	4 (80%)	24 (100%)	NS	NA	NA
Trimethoprim-sulfamethoxazole ^b^	1 (3.45%)	0 (0.0%)	1 (4.2%)	NS	NA	NA
Prior antibiotic usage—*n* (%)						
Third-generation cephalosporin	1 (3.45%)	0 (0.0%)	1 (4.2%)	NS	NA	NA
Macrolide	0 (0.0%)	0 (0.0%)	0 (0.0%)	NA	NA	NA

*ptxP*, pertussis toxin promoter; IQR, interquartile range; NA, not applicable; NS, not significant. ^a^ *p*-value was calculated with Mann–Whitney U test for parameters including white blood cells, lymphocytes, C reactive protein and length of stay. The *p* value was calculated with Fisher’s Exact Test for parameters including paroxysmal cough, fever, cyanosis, wheezing, pneumonia, respiratory failure, hospitalization, survival, oxygen therapy, mechanical ventilation and nasogastric feeding. ^b^ Result of this parameter is not available for all cases. ^c^ One patient did not receive treatment, while one patient received macrolide followed by trimethoprim-sulfamethoxazole due to treatment failure.

## References

[1] Ho F.W. (2025). Resurgence of Pertussis: A Global and Local Update.

[2] Gordon J.E., Hood R.I. (1951). Whooping cough and its epidemiological anomalies. Am. J. Med. Sci..

[3] Domenech de Cellès M., Rohani P. (2024). Pertussis vaccines, epidemiology and evolution. Nat. Rev. Microbiol..

[4] Yeung K.H.T., Duclos P., Nelson E.A.S., Hutubessy R.C.W. (2017). An update of the global burden of pertussis in children younger than 5 years: A modelling study. Lancet Infect. Dis..

[5] Centre for Health Protection of the Department of Health of the Hong Kong Special Administrative Region. Consensus Recommendations on Pertussis Vaccination for Pregnant Women in Hong Kong. https://www.chp.gov.hk/files/pdf/recommendations_on_pertussis_vaccination_for_pregnant_women_in_hk_formatted.pdf.

[6] Centre for Health Protection of the Department of Health of the Hong Kong Special Administrative Region. Recommendations on Updated Childhood Immunisation Programme Containing Inactivated Poliovirus and Acellular Pertussis Vaccines. https://www.chp.gov.hk/files/pdf/sas6_recommendation_on_updated_childhood_immunisation_programme_(dec2006).pdf.

[7] National Health Commission of the People’s Republic of China (2021). Childhood Immunization Schedule for National Immunization Program Vaccines—China (Version 2021). China CDC Wkly..

[8] Cai J., Chen M., Liu Q., Luo J., Yuan L., Chen Y., Chen M., Zeng M. (2023). Domination of an emerging erythromycin-resistant ptxP3 *Bordetella pertussis* clone in Shanghai, China. Int. J. Antimicrob. Agents.

[9] Jiang F., Ye X., Wang Y., Tang N., Feng J., Gao Y., Bao M. (2024). Factors associated with pregnant women’s willingness to receive maternal pertussis vaccination in Guizhou Province, China: An exploratory cross-sectional study. Hum. Vaccines Immunother..

[10] Guo M., Hu Y., Meng Q., Shi W., Yao K. (2024). Resurgence and atypical patterns of pertussis in China. J. Infect..

[11] Centers for Disease Control and Prevention, Center for Surveillance, Epidemiology and Laboratory Services, National Notifiable Diseases Surveillance System Pertussis (Week 49): Weekly Cases of Notifiable Diseases, United States, U.S. Territories, and Non-U.S. Residents Week Ending December 7, 2024. https://stacks.cdc.gov/view/cdc/174748.

[12] UK Health Security Agency. Confirmed Cases of Whooping Cough in England by Month. https://www.gov.uk/government/publications/pertussis-epidemiology-in-england-2024/confirmed-cases-of-pertussis-in-england-by-month.

[13] World Health Organization. Pertussis Reported Cases and Incidence. https://immunizationdata.who.int/global/wiise-detail-page/pertussis-reported-cases-and-incidence?CODE=Global&YEAR=.

[14] Fu P., Wang C., Tian H., Kang Z., Zeng M. (2019). *Bordetella pertussis* Infection in Infants and Young Children in Shanghai, China, 2016–2017: Clinical Features, Genotype Variations of Antigenic Genes and Macrolides Resistance. Pediatr. Infect. Dis. J..

[15] Fu P., Yan G., Li Y., Xie L., Ke Y., Qiu S., Wu S., Shi X., Qin J., Zhou J. (2024). Pertussis upsurge, age shift and vaccine escape post-COVID-19 caused by ptxP3 macrolide-resistant *Bordetella pertussis* MT28 clone in China. Clin. Microbiol. Infect..

[16] Fu P., Zhou J., Yang C., Nijiati Y., Zhou L., Yan G., Lu G., Zhai X., Wang C. (2023). Molecular Evolution and Increasing Macrolide Resistance of *Bordetella pertussis*, Shanghai, China, 2016–2022. Emerg. Infect. Dis..

[17] Bartkus J.M., Juni B.A., Ehresmann K., Miller C.A., Sanden G.N., Cassiday P.K., Saubolle M., Lee B., Long J., Harrison A.R. (2003). Identification of a Mutation Associated with Erythromycin Resistance in *Bordetella pertussis*: Implications for Surveillance of Antimicrobial Resistance. J. Clin. Microbiol..

[18] Ivaska L., Barkoff A.M., Mertsola J., He Q. (2022). Macrolide Resistance in *Bordetella pertussis*: Current Situation and Future Challenges. Antibiotics.

[19] Rodrigues C., Bouchez V., Soares A., Trombert-Paolantoni S., Aït El Belghiti F., Cohen J.F., Armatys N., Landier A., Blanchot T., Hervo M. (2024). Resurgence of *Bordetella pertussis*, including one macrolide-resistant isolate, France, 2024. Eurosurveillance.

[20] Zhang Q., Li M., Wang L., Xin T., He Q. (2013). High-resolution melting analysis for the detection of two erythromycin-resistant *Bordetella pertussis* strains carried by healthy schoolchildren in China. Clin. Microbiol. Infect..

[21] Yang Y., Yao K., Ma X., Shi W., Yuan L., Yang Y. (2015). Variation in *Bordetella pertussis* Susceptibility to Erythromycin and Virulence-Related Genotype Changes in China (1970–2014). PLoS ONE.

[22] Mooi F.R., van Loo I.H., van Gent M., He Q., Bart M.J., Heuvelman K.J., de Greeff S.C., Diavatopoulos D., Teunis P., Nagelkerke N. (2009). *Bordetella pertussis* strains with increased toxin production associated with pertussis resurgence. Emerg. Infect. Dis..

[23] Li L., Deng J., Ma X., Zhou K., Meng Q., Yuan L., Shi W., Wang Q., Li Y., Yao K. (2019). High Prevalence of Macrolide-Resistant *Bordetella pertussis* and ptxP1 Genotype, Mainland China, 2014–2016. Emerg. Infect. Dis..

[24] Zhou G., Li Y., Wang H., Wang Y., Gao Y., Xu J., Wang F., Peng T., Zhang M., Shao Z. (2024). Emergence of Erythromycin-Resistant and Pertactin- and Filamentous Hemagglutinin-Deficient *Bordetella pertussis* Strains—Beijing, China, 2022–2023. China CDC Wkly..

[25] Wu X., Du Q., Li D., Yuan L., Meng Q., Fu Z., Xu H., Yao K., Zhao R. (2022). A Cross-Sectional Study Revealing the Emergence of Erythromycin-Resistant *Bordetella pertussis* Carrying ptxP3 Alleles in China. Front. Microbiol..

[26] Centre for Health Protection of the Department of Health of the Hong Kong Special Administrative Region. Update on the Regional and Local Situations of Pertussis. https://www.chp.gov.hk/files/pdf/cdw_v20_5.pdf.

[27] (2019). Hospital Authority. HA Central Committee on Infectious Disaes and Emergency Respsonse. Fact Sheet on Perrtussis (Whooping cough). Internal Document. https://ha.home/ho/ps/FactSheetonPertussis.pdf.

[28] Tiwari T., Murphy T.V., Moran J. Recommended Antimicrobial Agents for the Treatment and Postexposure Prophylaxis of Pertussis: 2005 CDC Guidelines. https://www.cdc.gov/mmwr/preview/mmwrhtml/rr5414a1.htm.

[29] Communicable Diseases Network Australia. Pertussis CDNA National Guidelines for Public Health Units. https://www.health.gov.au/sites/default/files/2024-10/pertussis-whooping-cough-cdna-national-guidelines-for-public-health-units.pdf.

[30] UK Health Security Agency. Guidance on the Management of Cases of Pertussis in England During the Re-Emergence of Pertussis in 2024 Update: August 2024. https://assets.publishing.service.gov.uk/media/66c4a642808b8c0aa08fa7e7/UKHSA-guidance-on-the-management-of-cases-of-pertussis-during-high-activity-august-2024.pdf.

[31] Treatment of Pertussis. U.S. Centerse for Disease Control and Prevention. https://www.cdc.gov/pertussis/hcp/clinical-care/index.html.

[32] European Centre for Disease Prevention and Control. Laboratory Diagnosis and Molecular Surveillance of *Bordetella pertussis*. https://www.ecdc.europa.eu/sites/default/files/documents/bordetella-pertussis-laboratory-diagnosis-molecular-surveillance.pdf.

[33] Hill Bertha C., Baker Carolyn N., Tenover Fred C. (2000). A Simplified Method for Testing *Bordetella pertussis* for Resistance to Erythromycin and Other Antimicrobial Agents. J. Clin. Microbiol..

[34] Seven Treatments for Bacterial Sample Materials with MagNA Pure Bacteria Lysis Buffer. https://lifescience.roche.com/global/en/article-listing/article/seven-treatments-for-bacterial-sample-materials-with-magna-pure-.html.

[35] Wang Z., Cui Z., Li Y., Hou T., Liu X., Xi Y., Liu Y., Li H., He Q. (2014). High prevalence of erythromycin-resistant *Bordetella pertussis* in Xi’an, China. Clin. Microbiol. Infect..

[36] Tse H., Tsang A.K.L., Chu Y.-W., Tsang D.N.C. (2021). Draft Genome Sequences of 19 Clinical Isolates of Candida auris from Hong Kong. Microbiol. Resour. Announc..

[37] Bolger A.M., Lohse M., Usadel B. (2014). Trimmomatic: A flexible trimmer for Illumina sequence data. Bioinformatics.

[38] Bankevich A., Nurk S., Antipov D., Gurevich A.A., Dvorkin M., Kulikov A.S., Lesin V.M., Nikolenko S.I., Pham S., Prjibelski A.D. (2012). SPAdes: A new genome assembly algorithm and its applications to single-cell sequencing. J. Comput. Biol..

[39] Kolmogorov M., Raney B., Paten B., Pham S. (2014). Ragout-a reference-assisted assembly tool for bacterial genomes. Bioinformatics.

[40] Schouls L.M., van der Heide H.G., Vauterin L., Vauterin P., Mooi F.R. (2004). Multiple-locus variable-number tandem repeat analysis of Dutch *Bordetella pertussis* strains reveals rapid genetic changes with clonal expansion during the late 1990s. J. Bacteriol..

[41] (2023). WHO Director-General’s Opening Remarks at the Media Briefing. https://www.who.int/director-general/speeches/detail/who-director-general-s-opening-remarks-at-the-media-briefing---5-may-2023.

[42] Guo L., Zhang W., Su X., Huang H., Liu Y. (2018). Analysis on drug resistance of *Bordetella pertussis* isolated in Tianjin. Dis. Surveill..

[43] Zhang J.S., Wang H.M., Yao K.H., Liu Y., Lei Y.L., Deng J.K., Yang Y.H. (2020). Clinical characteristics, molecular epidemiology and antimicrobial susceptibility of pertussis among children in southern China. World J. Pediatr..

[44] Lin X., Zou J., Yao K., Li L., Zhong L. (2021). Analysis of antibiotic sensitivity and resistance genes of *Bordetella pertussis* in Chinese children. Medicine.

[45] Zhang J., Zhang D., Wang X., Wei X., Li H. (2022). Macrolide susceptibility and molecular characteristics of *Bordetella pertussis*. J. Int. Med. Res..

[46] Li J., Liu L., Zhang H., Guo J., Wei X., Xue M., Ma X. (2023). Severe problem of macrolides resistance to common pathogens in China. Front. Cell. Infect. Microbiol..

[47] Ma E.S., Hsu E., Chow V., Chow T., Kung K.H., Au A., Chen H. (2025). Rebound of Antibiotic Use and Respiratory Infections After Resumption of Normalcy From COVID-19 in Hong Kong. Infect. Drug Resist..

[48] Mi Y.M., Hua C.Z., Fang C., Liu J.J., Xie Y.P., Lin L.N., Wang G.L. (2021). Effect of Macrolides and β-lactams on Clearance of *Bordetella pertussis* in the Nasopharynx in Children With Whooping Cough. Pediatr. Infect. Dis. J..

[49] Petersen R.F., Dalby T., Dragsted D.M., Mooi F., Lambertsen L. (2012). Temporal trends in *Bordetella pertussis* populations, Denmark, 1949–2010. Emerg. Infect. Dis..

[50] Shi W., Meng Q., Hu Y., Yao K. (2024). Modifying antibiotic treatment strategies in the face of pertussis surge associated to erythromycin resistance in China. J. Infect..

[51] Gordon K.A., Fusco J., Biedenbach D.J., Pfaller M.A., Jones R.N. (2001). Antimicrobial susceptibility testing of clinical isolates of *Bordetella pertussis* from northern California: Report from the SENTRY Antimicrobial Surveillance Program. Antimicrob. Agents Chemother..

[52] Kimberlin D.W., Barnett E.D., Lynfield R., Sawyer M.H., Committee on Infectious Diseases, American Academy of Pediatrics (2021). Tetracyclines. Red Book: 2021–2024 Report of the Committee on Infectious Diseases.

[53] Kimberlin D.W., Barnett E.D., Lynfield R., Sawyer M.H., Committee on Infectious Diseases, American Academy of Pediatrics (2021). Fluoroquinolones. Red Book: 2021–2024 Report of the Committee on Infectious Diseases.

[54] Koide K., Yao S., Chiang C.S., Thuy P.T.B., Nga D.T.T., Huong D.T., Dien T.M., Vichit O., Vutthikol Y., Sovannara S. (2022). Genotyping and macrolide-resistant mutation of *Bordetella pertussis* in East and South-East Asia. J. Glob. Antimicrob. Resist..

[55] Mei Z., Zhujun S., Wenhong Z., Jun X. (2024). Guidelines for diagnosis and management and prevention of pertussis of China (2024 edition). Chin. Med. Assoc..

[56] Feng Y., Chiu C.-H., Heininger U., Hozbor D.F., Tan T.Q., von König C.-H.W. (2021). Emerging macrolide resistance in *Bordetella pertussis* in mainland China: Findings and warning from the global pertussis initiative. Lancet Reg. Health–West. Pac..

[57] Hong Kong Tourism Board. Monthly Report—Visitor Arrival Statistics: Jan 2024. https://www.discoverhongkong.com/content/dam/dhk/intl/corporate/newsroom/tourisum-statistics/2024/tourism_stat_01_2024.pdf.

[58] Centre for Health Protection of the Department of Health of the Hong Kong Special Administrative Region. Number of Notifiable Infectious Diseases by Month. https://www.chp.gov.hk/en/statistics/data/10/26/43/7060.html.

[59] Jenkinson D. (2019). Pertussis (whooping cough) is common in teens and adults. BMJ.

